# Complete mitochondrial genome sequence of *Hemiculter bleekeri*

**DOI:** 10.1080/23802359.2016.1186506

**Published:** 2016-12-09

**Authors:** Yandong Niu, Xiaoli Wu, Canming Zhang, Ying Wei

**Affiliations:** a Hunan Academy of Forestry, Changsha, China;; b Hunan Dongting Lake Wetland Ecosystem Research Station, Yueyang, China;; c Hunan Cooperation Center of Water Resources Research and Development, Changsha, China;; d Forestry Department of Hunan Province, Changsha, China

**Keywords:** Base composition, *Hemiculter bleekeri*, mitogenome, phylogenetic

## Abstract

In our research, the complete mitochondrial DNA (mtDNA) of *Hemiculter bleekeri* was obtained from the dorsal myotome of the fish. The total length of the mitochondrial genome is 16,617 bp and deposited in GenBank with accession number KU198332. The gene arrangement was similar to other bony fishes, which contained 37 genes (13 protein-coding genes, 2 ribosomal RNA and 22 transfer RNAs) and a major non-coding control region. The mtDNA may provide some important genetic background information of this valuable fish. The G contents was relatively low in the total mtDNA with the GCskewthinsp;= −0.22. The negative GC-skew ranging from −0.51(ATP8) to −0.19(CO1), and the more positive AT-skew varying from −0.26(ATP8) to −0.03(CO1). The phylogenetic analysis demonstrated that *H. Bleekeri* was clustered together with *Hemiculter leucisculus,* which could be a sister species.

With a suitable marker, the reconstruct of the evolutionary process may be more realistic. Mitochondria are double-layered membrane-bound organelles, found in nearly all eukaryotic cells with an estrangement genetic affinity. In addition, the mitochondria have some unique characteristics, such as small size (16K), high abundance, maternal inheritance, lack of recombination and rapid evolutionary rate (Chen et al. 2012). It has been considered as a natural marker and widely applied in population genetics and evolutionary studies (Rand 2008; Su et al. 2014).

*Hemiculter bleekeri* was assigned to Order Cypriniformes; Family Cyprinidae and genus *Hemiculter*, which was obtained from the Dongting lake, Hunan, China (111.42°E 29.15°N). In this study, the dorsal myotome of the fish was obtained and preserved in 95% ethanol. The Blood & Cell Culture DNA Kit (QIAGEN, Düsseldorf, Germany) was used to extract the genomic DNA. It was then stored at −20 °C until further use. For a better understanding of the characteristic and the evolutionary relationship, we focused mainly on the genetic information contained in the complete mitochondrial genomes of the fish.

The mitochondrial genome has been deposited in GenBank with accession number KU198332. The total length of the complete mitochondrial genome was 16,617 bp in length which included 13 protein-coding genes, 2 ribosomal RNA (rRNA) genes, 22 transfer RNA (tRNA) genes and one control region (D-loop). The gene arrangement and transcriptional direction were similar to those seen in typical teleosts mitogenomes (Li et al. 2010; Chen et al. 2012). Most genes were encoded on the H-strand, except for ND6 gene and eight tRNA genes that were encoded on the L-strand. All the 13 protein-coding genes contain the same start codon ATG except the gene CO1, which contains GTG instead. However, the termination codons of the 13 protein-coding genes are varied, five genes (ATP6, CO1, ND4L, ND2, ND6) ended with TAA, five (CO2, CYTB, ND1, ND3, ND4) with T–, CO3 stopped with TA-, while ATP8 and ND5 stopped with TAG.

With an estrangement genetic affinity, 22 tRNA genes were identified. The 22 tRNA genes can be divided into three tRNA clusters, which were IQM (tRNA-Ile, tRNA-Glu and tRNA-Met), WANCY (tRNA-Trp, tRNA-Ala, tRNA-Asp, tRNA-Cys and tRNA-Tyr), and HSL (tRNA-His, tRNA-Ser and tRNA-Leu) and other single tRNA.

As observed in other bony fish, the G contents were the lowest (17.54%) (Oh et al. 2007). The AT-skew and GC-skews always show high inter or intra-phylum variation, which might affect phylogenetic analyses (Nesnidal et al. 2011). The nucleotide skewness for the coding strands of *H. bleekeri* (GC-skew = −0.22, AT-skew = 0.07) is biased towards A and C. A similar trend has been observed in other teleost mitogenomes: the negative GC-skew ranging from −0.51 (ATP8) to −0.19 (CO1), while the more positive AT-skew varying from −0.26 (ATP8) to −0.03 (CO1).

Phylogenetic analysis was performed using the mitochondrial genomes of 14 fish species from the Cyprinidae (Figure 1). Each of the datasets was aligned using Clustal X and analyzed by neighbour-joining (N-J) in MEGA 4.0 and bootstrap analysis was performed with 1000 replications. The result demonstrated that the *H. Bleekeri was* clustered together with *Hemiculter leucisculus*, which could be a sister species. Then, the *H. Bleekeri* clustal together with *Plagiognathops microlepis* which belong to Cyprinidae, Plagiognathops.

**Figure 1.**
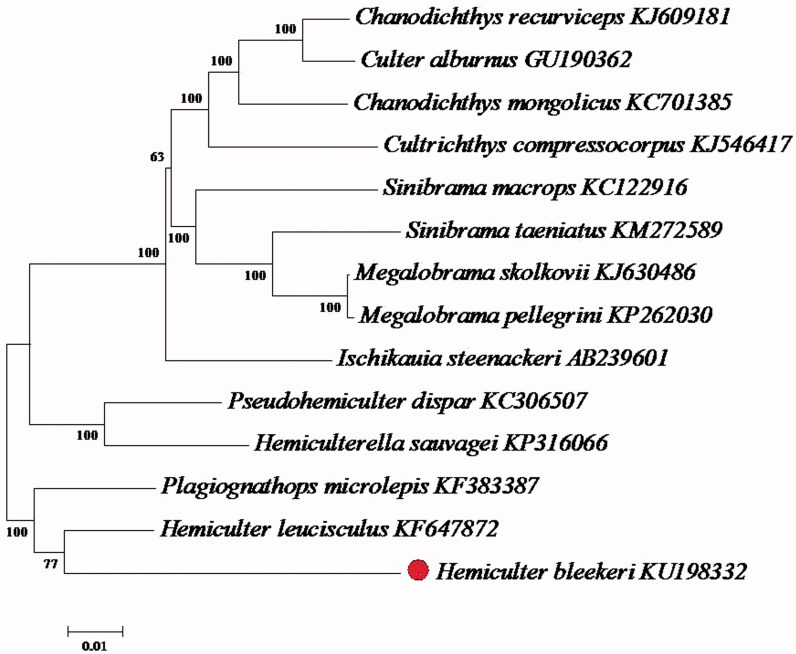
Phylogenetic analysis based on mitochondrial genome sequences with maximum-likelihood distance (M-P) method.
